# Value of Elastography in Differentiating Benign from Malignant Breast Lesions Keeping Histopathology as Gold Standard

**DOI:** 10.7759/cureus.5861

**Published:** 2019-10-08

**Authors:** Faryal Farooq, Syed Mubarak, Shaista Shaukat, Noman Khan, Kahkashan Jafar, Tariq Mahmood, Muhammad Arif Saeed

**Affiliations:** 1 Diagnostic Radiology, Jinnah Post Graduate Medical Centre, Karachi, PAK; 2 Diagnostic Radiology, Karachi Medical and Dental College, Karachi, PAK; 3 Radiology, Jinnah Post Graduate Medical Centre, Karachi, PAK; 4 Radiology, Aga Khan University Hospital, Karachi, PAK; 5 Radiology, Abbasi Shaheed Hospital, Karachi, PAK

**Keywords:** breast cancer, breast elastography, breast malignancy, breast ultrasound

## Abstract

Background: Breast cancer is the most common cancer in females, both in developed and developing countries. Pakistan has the highest breast cancer incidence rate in Asia. Guidelines recommend screening for detecting breast cancer with mammography and ultrasonography (US). Shear-wave elastography (SWE) is a newer technique that can aid additional characterization of breast lesions.

Objective: The aim of this study was to determine the diagnostic accuracy of breast ultrasound elastography in differentiating benign from malignant breast lesions using histology diagnosis as the gold standard.

Materials and methods: The study was conducted at the Abbasi Shaheed Hospital and Jinnah Post Graduate Medical Centre, Karachi. All consecutive patients undergoing breast biopsy and elastography of breast lesions were enlisted; 2 x 2 tables were used to measure the sensitivity, specificity, positive predictive value (PPV), negative predictive value (NPV), and diagnostic accuracy of breast ultrasound elastography for differentiation of benign from malignant breast masses.

Results: A total of 155 female patients were included with a mean age of 45.41 ± 14.24 years (range 20-70 years). On histological evaluation, 115 (74.2%) lesions were malignant and 40 (25.8%) were benign. The overall average mean elastography value was 108.45 kPa ± 52.75. The mean elastography (E_Mean_) value for benign breast lesions was 48.96 kPa ± 42.32 and 132.78 kPa ± 42.32 for malignant lesions. The difference in mean elastography values of benign and malignant breast lesions was statistically significant (48.96 kPa ± 42.32 vs 32.78 kPa ± 42.32, P <0.001). The area under the curve (AUC) was 0.952, optimal cutoff E_Mean_ value of 72 kPa and higher likelihood ratio was 9.41. A cutoff mean elastography (E_Mean_) value of ≤ 72 kilopascal (kPa) for benign lesions had sensitivity 92.17%, specificity 90.4%, PPV 96.36%, NPV 80.0% and diagnostic accuracy 91.61%.

Conclusion: Ultrasound elastography was found to have high sensitivity and specificity and diagnostic accuracy for differentiating benign from malignant breast lesions. Use of shear-wave elastography may increase malignancy detection rate by reducing the need for biopsy in benign breast lesions.

## Introduction

Breast cancer is the most common cancer diagnosed in women globally and the second most common malignancy overall, after lung cancer [1-2]. The incidence of breast cancer has been on the rise over the last few decades. The incidence of breast cancer is the highest in Pakistan (50.1/100,000) [3]. Data collected from 1995-1997 shows that breast cancer accounts for almost one-third of all female cancers in Karachi [3]. With more than half the population at risk, the incidence of breast cancer has alarmingly increased over the last few decades [4]. Breast cancer is uncommon before age 25 but the risk steadily increases with age, doubling every 10 years until menopause and slows dramatically afterwards [5].

Mammography is a valuable modality for screening in breast cancer however has low sensitivity in dense breast tissue [6-7]. The sensitivity of mammography in breast cancer is reduced from an overall 85% to 47.8%-64.4% in dense breast tissue [8]. Breast ultrasound is another diagnostic modality available for the evaluation of breast lesions and is common in clinical practice due to its higher sensitivity. There is some advocacy for breast ultrasound to be the primary imaging modality in the evaluation of breast lesions [9]. The Breast Imaging Reporting and Data System (BIRADS) along with ultrasonographic (US) descriptors are used to characterize and categorize breast lesions [10]. Breast ultrasound has its limitations; limited ability to distinguish isoechoic lesions from surrounding fat, inability to image areas deep within the breast, and poor detection of microcalcifications [11]. 

Ultrasound elastography is a newer modality which assesses the tissue differences regarding stiffness or elasticity of lesions that were, historically assessed by palpation [12]. Elastography was first introduced in 1990 and entered clinical practice in 1997 [13-14]. 

Elastography is a non-invasive imaging technique in which local tissue strains are measured directly or indirectly by application of external stress. The tissue displacement is measured and a calculation of tissue stiffness is made based on tissue displacement [12]. Shear-wave elastography (SWE) reduces operator dependency which was encountered previously in free hand elastography [15]. A quantitative assessment of viscoelastic properties of tissue is obtained by inducing mechanical vibrations through a focused beam, which is expressed as Young’s modulus or displayed as a color overlay of the lesion [16]. SWE is reported to have excellent diagnostic performance in distinguishing benign breast masses from malignant lesions [17].

SWE is gaining popularity in clinical practice in Pakistan; however there is scarcity of published literature from local institutions [18]. The purpose of this study was to determine the diagnostic accuracy of breast ultrasound elastography in distinguishing benign breast lesions from malignant breast lesions keeping histopathological diagnosis as the gold standard.

## Materials and methods

The dual center study prospective study was conducted at the Abbasi Shaheed Hospital and Jinnah Post Graduate Medical Centre, from June 2017 to February 2018. Approval for the study was obtained from institutional Ethical Review Committee of Jinnah Post Graduate Medical Centre. Informed consent was obtained from all patients prior to their enrollment in the study.

All females between the ages of 20 and 60 referred for evaluation of palpable breast lumps were included. Patients who had cystic breast lesions, post surgical or chemoradiation status, the clinical picture of infection or breast abscess were excluded from the study. Patients were also excluded if they had breast implants, had undergone a prior biopsy of breast lesions under examination or the lesion was subcentimeter. The final study population consisted of 155 patients.

US elastography was performed and interpreted by two experienced consultant radiologists with more than five years of post-fellowship experience. On a predefined proforma, the clinical history and physical examination findings were recorded. 

A dedicated breast ultrasound was performed with Aixplorer (SuperSonic Imagine, Aix-en-Provence, France). The lesions were classified on gray scale ultrasound and color doppler imaging as BIRADS US category 2, category 3, category 4 and category 5. SWE of all lesions was performed using multi-Q box elastography and mean elastography (E_Mean_) value was recorded on a predefined proforma along with clinical history, grayscale ultrasound findings, and subsequently histopathological diagnosis. All lesions were subjected to gray-scale ultrasound, color doppler imaging and multi-Q box elastography values were evaluated. The mean elastography (E_Mean_) value was recorded for each lesion. The lesions were classified to individual categories of BIRADS category 2, category 3, category 4 and category 5. BIRADS category 1 lesions were excluded from the study. All lesions from BI-RADS US II-V were subjected to histopathology. 

The range of elastography values was plotted and mean elastography (E_Mean_) values were calculated for all lesions. A cutoff mean elastography (E_Mean_) value of ≤ 72 kPa was set for benign lesions and ≥ 102 kPa for malignant lesions. Lesions with mean elastography (E_Mean_) values between 72-102 (kPa) were suspected to be malignant. 

The statistical analysis was performed using free statistical software package R (version 3.5.2) [19]. Using 2 x 2 tables, the sensitivity, specificity, positive predictive value, negative predictive value, and diagnostic accuracy were determined. Receiver operator characteristics (ROC) curve were also determined. The area under the ROC curve (AUC) analysis was performed to determine the optimal cutoff value of mean elastography value. A two-tailed p-value <0.05 was considered statistically significant.

## Results

The final study sample comprised of 155 women with a mean age of 45.41 ± 14.24 years (range 20-70 years). The overall mean elastography value (E_Mean_) was 108.45 kPa ± 52.75. The mean elastography value was 48.96 kPa ± 42.32 for benign lesions and 132.78 kPa ± 42.32 for malignant lesions, with statistically significant difference in the mean elastography values between the benign and malignant lesions (P<0.001). 

On histopathological evaluation, 115 (74.2%) were found to be malignant and 40 (25.8%) were benign. On the basis of elastography values, 110 lesions were labelled as malignant or suspicious of malignancy and 45 were labelled as benign (Table 1). Based on the BIRADS US assessment, 34 (21.9%) lesions were labelled as benign and 121 as malignant or suspicious of malignancy.

**Table 1:** Comparison of histopathological diagnosis with elastography BIRADS: Breast Imaging Reporting and Data System.

Table 1: Comparison of histopathological diagnosis with Elastography					
Elastography	Histopathology Diagnosis (BIRADS US Assessment)			P-value	Kappa Value
	Positive (III-V)	Negative (II)	Total		
Positive	106(68.4%)	4(2.6%)	110(71%)	<0.001*	0.79
Negative	9(5.8%)	36(23.2%)	45(29%)		
Total	115(74.2%)	40(25.8%)	155(100%)		

**Figure 1:**
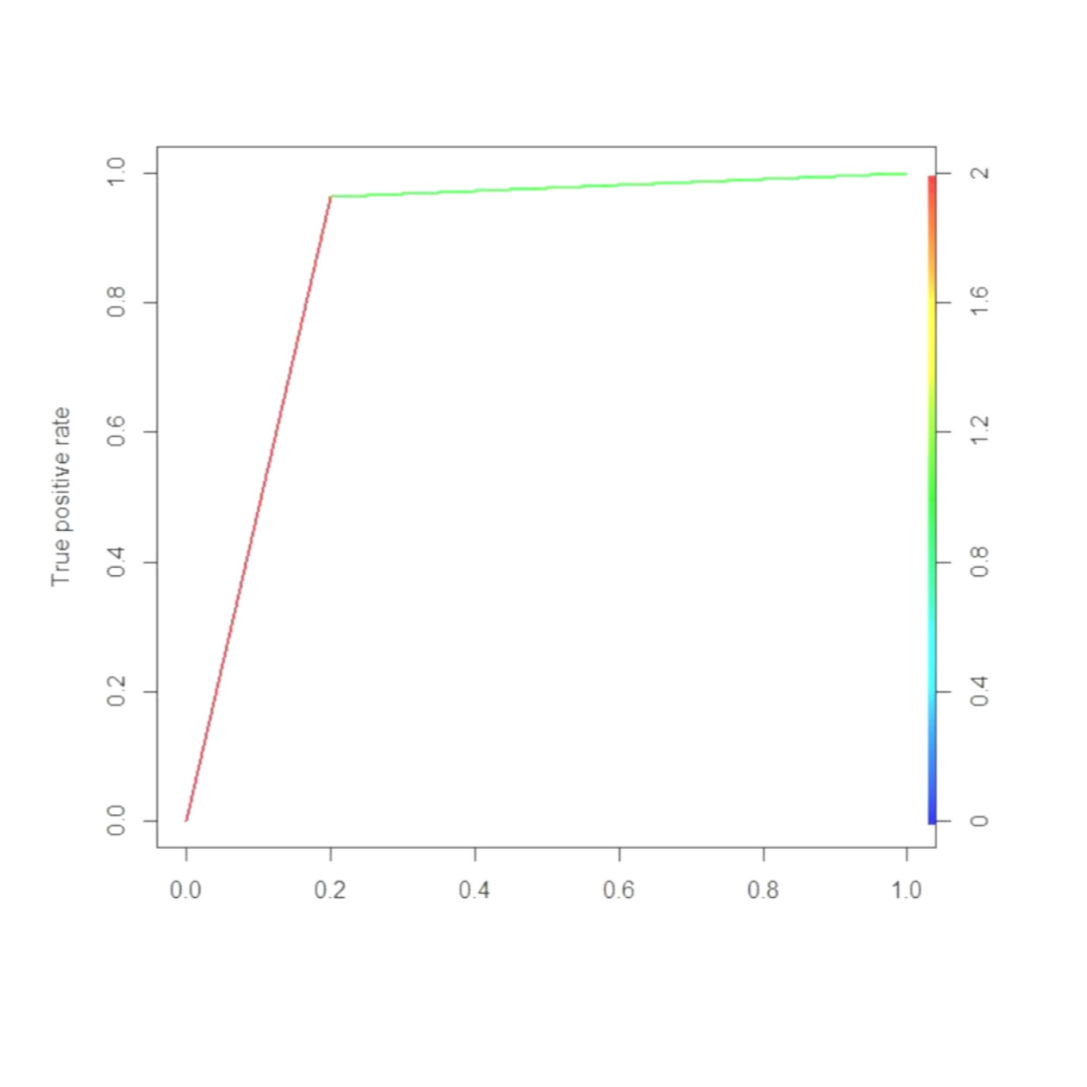
Receiver operator characteristics (ROC) curve

**Figure 2:**
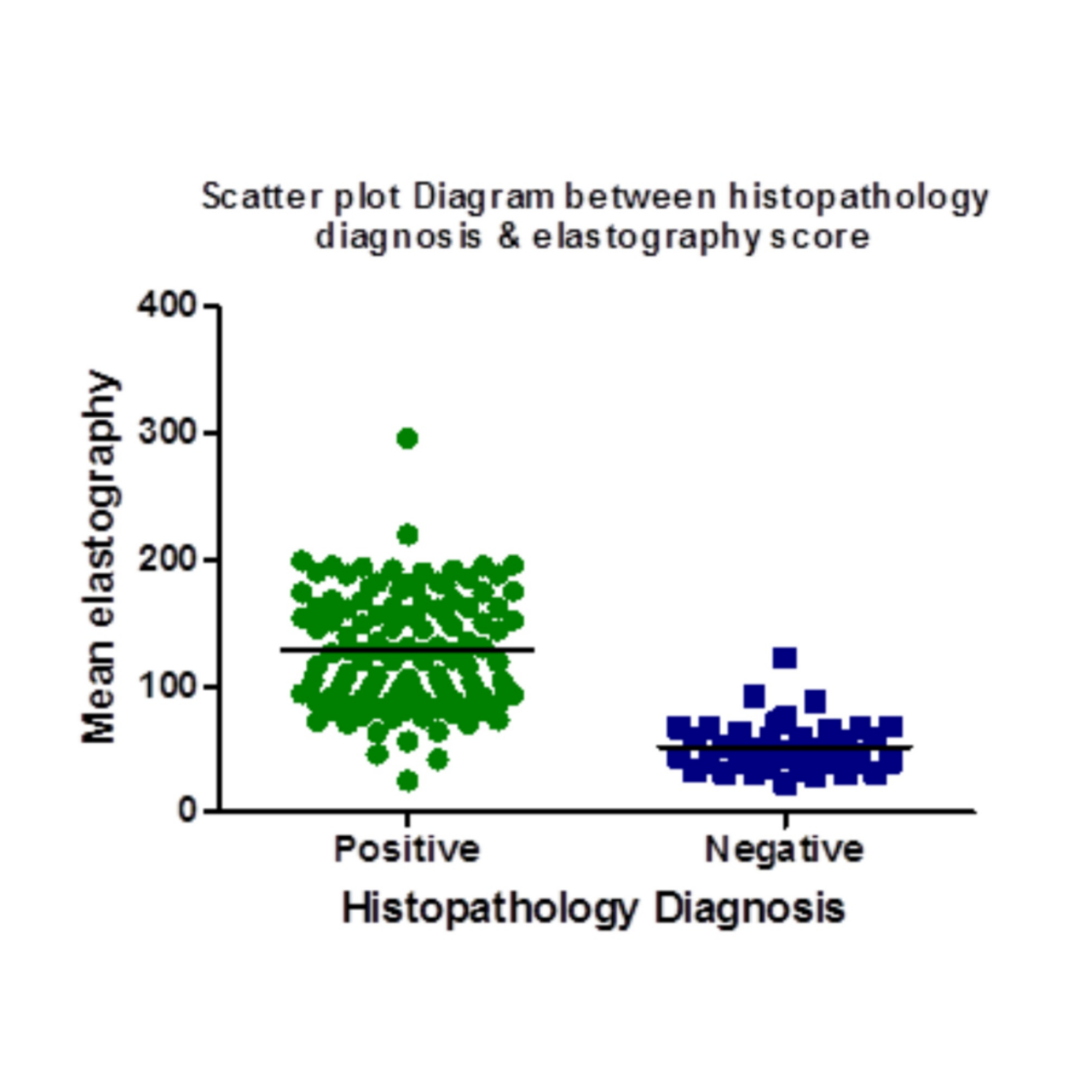
Scatter plot diagram between histopathology diagnosis & elastography score value

**Figure 3:**
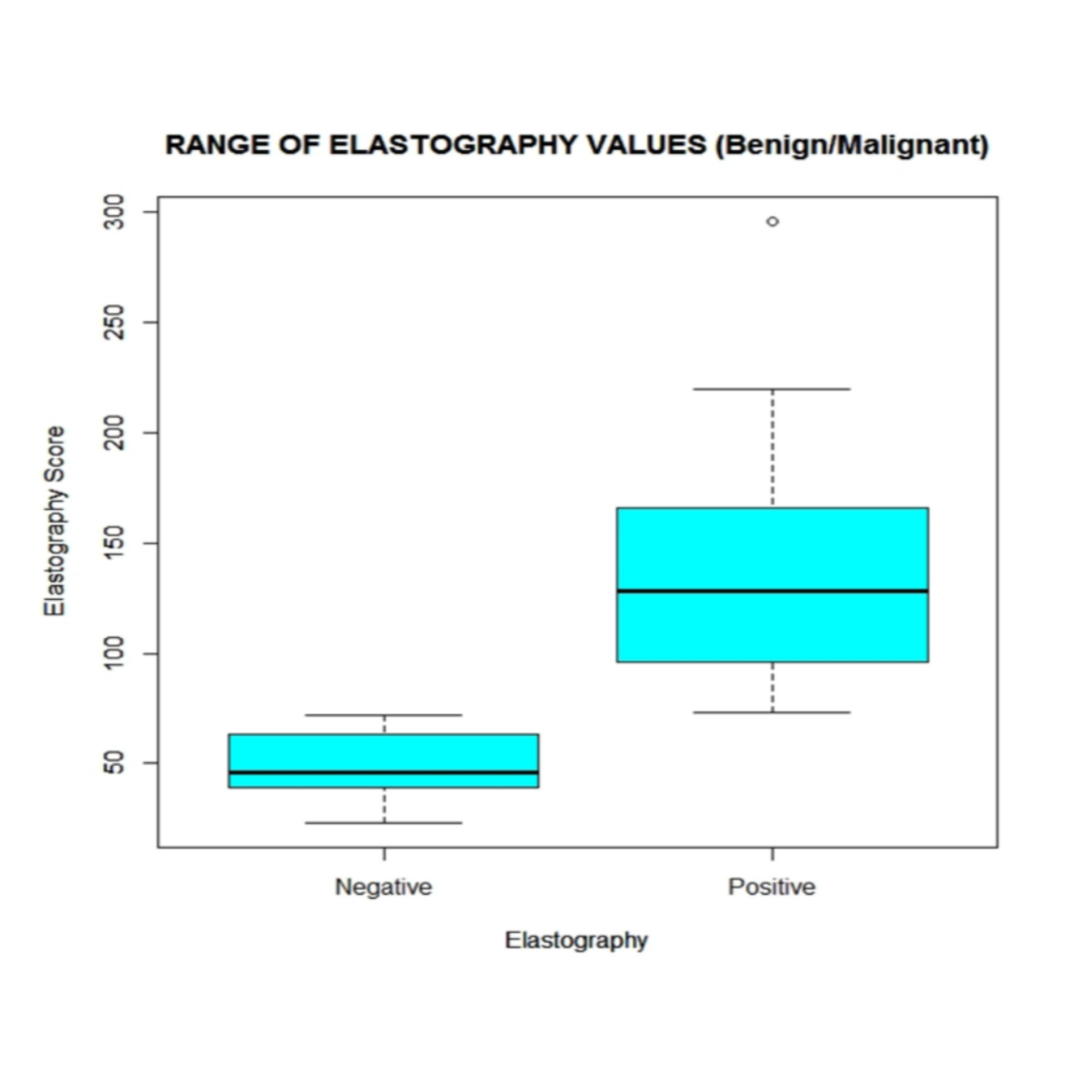
Whisker’s Box plot of range of elastography values (benign/malignant)

SWE had sensitivity 92.17%, specificity 90.4%, PPV 96.36% and NPV 80.0% and an overall diagnostic accuracy 91.61% (95% confidence Interval, 86.08% to 95.46%; P= <0.001) in diagnosing benign breast lesions (Table 2). The area under the curve was 0.952 (95% Confidence Interval, 0.916 to 0.9879; P= <0.01), the AUROC cutoff < 72 and higher likelihood ratio was 9.41 (Figures 1-3).

**Table 2:** Diagnostic accuracy of breast ultrasound elastography score

Table 2: Diagnostic accuracy of breast ultrasound elastography score					
Cutoff	Sensitivity (95% Confidence Interval)	Specificity (95% Confidence interval)	Likelihood Ratio	Area (95% Confidence interval)	P-value
≤ 72	92.17 (85.66% to 96.36%)	90.43 (83.53% to 95.13%)	9.41	0.952 (0.916 to 0.9879)	<0.001

## Discussion

While elastography imaging has been under evaluation for breast imaging, there has been a renewed focus in recent years. The main disadvantages of static elastography were the inability to provide a quantitative assessment and significant interobserver variability [20]. The main advantage of SWE over conventional elastography is its higher reproducibility and objectivity, as it allows to "move" the tissue by itself without the aid of external compression by transducer [21]. Many shear wave electrographic parameters can be used for the assessment of breast lesions related to elasticity values, i.e., minimum (E_Min_), mean (E_Mean_), and maximum (E_Max_). EMin, E_Mean_, and E_Max_ represent the stiffness of the lesion whereas E_Ratio_ represents the relative stiffness of the lesion to fat tissue which has a coherent elasticity value (3 kPa) [22]. Published literature suggests a higher sensitivity of Emax and higher specificity of Eratio parameters [17,23-24].

The reproducibility of SWE parameters as a useful biomarker for differentiation of benign and malignant breast lesions has been variable in the published literature with reported sensitivity and specificity of SWE ranging from 70.1%-98.6%, and 45.7%-98.5% respectively [20,23]. With an overall sensitivity of 92%, our initial experience with SWE has shown that it is useful in the characterization of benign breast lesions. Our study results are similar to other published studies. Berg et al. in a large multinational study demonstrated that elastography could reduce unnecessary biopsies of low‐suspicion category 4a masses [25].

Our results are similar to Athanasiou et al. who reported a mean elasticity value of 45.3 kPa for benign lesions and 146.6 kPa for malignant lesions [26]. The mean elastography value in our study was 48.96 kPa for benign lesions and 132.78 kPa for malignant lesions. The difference in the mean elastography values of benign and malignant lesions was statistically significant (P<0.001).

BIRADS US Category 3 lesions are more frequently malignant than non-palpable lesions in this category, this was also noted in our study [27]. The most common age of patients with malignant lesions was between 40 and 60 years. This is consistent with prior studies as the prevalence of breast cancer is highest in this age group [5]. 

**Figure 4:**
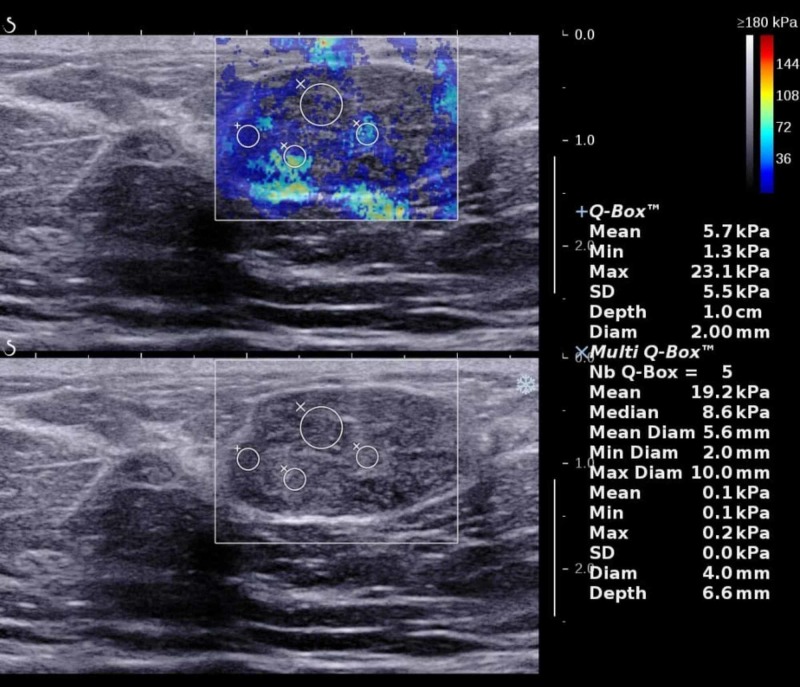
Breast lesion with low mean elastography value, which was diagnosed as fibroadenoma

Fibroadenoma is a common benign tumor, resulting from an excess proliferation of connective tissue and is often subjected to percutaneous biopsy [28]. A study by Masroor et al. showed that 80% of all biopsied breast lesions were benign in the Pakistani population [29]. This is similar to other studies, that have reported a cancer detection rate of only 10%-30% on breast biopsy [17]. Biopsy of a benign lesion causes physical and emotional discomfort along with increased cost. In a country where obtaining breast biopsy is limited to a few centers, the overwhelming rate of benign results on breast biopsy can be reduced by the use of elastography. An example of fibroadenoma is shown below, with low E_Mean_ value of 5.7 kPa (Figure 4). 

There are limitations of our study. In our study, 74% of breast lesions biopsied were malignant on histological evaluation. This high malignancy detection rate is not in keeping with local and international studies. This may be explained by a small sample size and possible subject selection bias. A larger study is therefore being planned for future research. 

Early diagnosis and treatment of breast cancer are crucial for better prognosis. Biopsy of breast lesion provides definitive diagnosis however dedicated breast biopsy facilities in Pakistan cannot keep up with the increasing demand [30]. Therefore, recent developments in elasticity imaging may be applied in the clinical setting for reliable characterization of benign breast lesions. Breast elastography may reduce biopsy of benign lesions and increase the time required for follow-up, translating into increased malignancy detection rate. Although further research is necessary, our initial results are promising.

## Conclusions

BIRADS assessment is improved by SWE in differentiating benign from malignant breast lesions. A cutoff mean elastography (E_Mean_) value of ≤ 72 kPa is highly sensitive and specific for characterizing benign lesions. Use of shear-wave elastography may increase malignancy detection rate by reducing the need for biopsy in benign breast lesions.
